# Creatinine Normalization Over-Corrects Urinary Electrolytes but Not Protein: Implications for Hydration Monitoring in Athletes

**DOI:** 10.3390/metabo16080548

**Published:** 2026-08-02

**Authors:** Ghareeb O. Alshuwaier

**Affiliations:** Department of Exercise Physiology, College of Sport Sciences and Physical Activity, King Saud University, Riyadh 11451, Saudi Arabia; galshuwaier@ksu.edu.sa

**Keywords:** hydration assessment, urine specific gravity, spot urine, electrolytes, creatinine, metabolomics, heat, football

## Abstract

**Background**: Creatinine normalization is the default adjustment for urine dilution in spot-urine monitoring of athletes, yet its appropriateness is seldom tested in field data. We tested whether it removes or introduces concentration dependence across a panel of urinary analytes in soccer players training in extreme heat and whether raw versus normalized values change the inferred response. **Methods**: Sixteen male soccer players (age 19.9 ± 0.8 y) provided spot urine at three daily time points (morning, pre-training, post-training) over six training days in Riyadh, Saudi Arabia. Sodium, potassium, chloride, calcium, magnesium, glucose, urea nitrogen, uric acid, total protein, and creatinine were measured. For each analyte we quantified its scaling exponent with creatinine, the correlation of raw and normalized values with creatinine, and the within-day pattern on raw versus normalized values using linear mixed-effects models. **Results**: Urinary creatinine varied 22.7-fold (coefficient of variation 39%). Only protein and glucose scaled proportionally to creatinine (exponents ≈1.0) and normalized cleanly (ratio–creatinine r = −0.06 and −0.21). The remaining seven analytes, including all electrolytes, scaled weakly (exponents 0.16–0.49), so normalization over-corrected them, producing strong negative ratio–creatinine correlations (uric acid −0.74, sodium −0.59, chloride −0.55). For four of nine a the inferred within-day response differed in direction or significance between raw and normalized values; restricting creatinine to the recommended 0.3–3 g·L^−1^ range attenuated but did not abolish the over-correction. **Conclusions**: Creatinine normalization suited protein and glucose but over-corrected every electrolyte and could reverse inferred responses. In this setting of hot-environment team-sport athletes, creatinine normalization should not be assumed adequate for electrolytes, which instead warrant interpretation against an independent index of urine concentration such as osmolality or specific gravity.

## 1. Introduction

Association football is an intermittent, high-intensity sport in which outfield players typically cover 10–12 km per match, interspersed with frequent sprints, accelerations, and decelerations [1]. This activity generates a substantial metabolic heat load that is dissipated chiefly through evaporative sweating, and when training or competition takes place in hot environments, sweat rates rise steeply and players can accrue large fluid and electrolyte deficits within a single session [2]. Such conditions are the norm across the Arabian Peninsula and other hot regions where the sport is expanding and where major international tournaments are increasingly staged, so that the assessment of hydration and electrolyte status has become a routine and consequential concern for practitioners working with these athletes [3].

Decisions about when and how much athletes should drink, whether they are ready to train or compete, and whether they are at risk of heat illness increasingly rest on objective markers of hydration and electrolyte status [4,5,6]. In the field, urine is the medium of choice for these markers because it can be obtained non-invasively, inexpensively, and repeatedly across a training day [7]. This convenience carries an interpretive cost: the concentration of any solute in a spot-urine sample reflects not only how much of that solute the kidney is handling but also how much water happens to accompany it, and urine flow varies several-fold with hydration, fluid intake, and exercise [8]. Because such markers inform real decisions, error introduced at the measurement stage is not merely statistical noise; it can systematically misrepresent an athlete’s physiological state.

The standard remedy is to normalize each analyte to urinary creatinine and to report an analyte-to-creatinine ratio. Creatinine is generated by muscle metabolism and excreted at a broadly stable daily rate, and on this basis, it is used almost reflexively as an internal index of urine dilution throughout clinical chemistry, biomonitoring, and sports science [9,10]. It is likewise the most widely used dilution correction in urinary metabolomics, where it is applied alongside osmolality- and signal-based alternatives such as probabilistic quotient normalization [11,12,13,14]. The appeal is obvious: a single, routinely measured solute appears to convert an otherwise uninterpretable spot concentration into a flow-independent estimate of excretion.

That appeal rests on two assumptions, each of which is frequently violated. The first is that creatinine excretion is constant within and between individuals; in reality it varies with muscle mass, dietary protein intake, sex, and glomerular filtration rate and can shift acutely with urine flow itself [10,15,16]. The second, and less widely appreciated, is that the analyte of interest is handled by the kidney in the same flow-dependent manner as creatinine, only then does dividing one by the other cancel the influence of dilution. When solutes scale with urine concentration differently from creatinine, the ratio does not become flow-independent but instead retains, or even acquires, a systematic dependence on concentration, so that the intended correction introduces a distortion of its own [15,16]. In athletes, even a difference in lean mass has been shown to bias creatinine-referenced hydration indices [17].

These conditions for failure converge in the very population in which urinary monitoring is most heavily used: athletes exercising in the heat. Here urine concentration can swing widely within a single day as players move between rested, pre-exercise, and dehydrated post-exercise states. The analytes of greatest practical interest, the electrolytes, are lost in substantial quantities in sweat [2,18,19] and are subject to tight, hormonally driven renal regulation that need not parallel the handling of creatinine [20]; indeed, established equations that estimate daily sodium excretion from spot urine themselves rest on creatinine-based assumptions [21,22]. The most common urinary hydration index, specific gravity, already has contested validity as a marker of fluid status [23,24,25,26,27]. The correction is therefore most likely to mislead precisely where it is most relied upon. The direction of this error has practical importance: because urine becomes more concentrated as an athlete dehydrates, a creatinine-referenced electrolyte value tends to fall precisely when true electrolyte and fluid losses are greatest, so a dehydrating player may appear, on paper, to be conserving electrolytes normally, a misclassification that could delay or misdirect rehydration.

The stakes of this measurement problem extend well beyond a single sport. Creatinine-referenced ratios underpin field hydration monitoring, population estimates of dietary sodium intake from spot urine, occupational and environmental biomonitoring, and biomarker discovery in urinary metabolomics [10,11,21]; in each of these applications, a correction that behaves differently for different analytes could distort the comparisons drawn. Yet in applied practice, creatinine normalization is typically applied as an unexamined default: it is seldom demonstrated in real field data which analytes the correction genuinely rescues, which it distorts, and, most consequentially, whether the choice between raw and normalized values changes the conclusion an investigator or practitioner would reach.

Addressing this does not require further validation against an assumed-correct reference, but a direct interrogation of the correction itself, using data in which urine concentration varies over a wide range within the same individuals. Repeated spot-urine sampling from soccer players training in the extreme natural heat of Riyadh, Saudi Arabia, spanning a rested morning void, a pre-training sample, and a post-training sample across successive days, generates exactly such a range of concentrations and so provides a naturally occurring test bed for the question.

Accordingly, the present study set out, in field spot-urine samples from male soccer players training in extreme natural heat, to: (i) quantify the magnitude of within- and between-day variation in urine concentration, indexed by creatinine; (ii) determine, across a panel of urinary analytes, the extent to which creatinine normalization removes, or conversely introduces, a dependence on urine concentration; and (iii) establish whether the choice between raw and creatinine-normalized values alters the inferred within-day and across-day response. In doing so, the study aimed to identify, for an ecologically valid athletic setting, the analytes for which creatinine normalization is appropriate and those for which it is misleading.

## 2. Materials and Methods

### 2.1. Participants

Sixteen male soccer players from a single professional squad volunteered to participate (age 19.9 ± 0.8 y; body mass 65.6 ± 5.0 kg; stature 1.74 ± 0.04 m; body mass index 21.1 ± 1.4 kg·m^−2^). All were engaged in regular structured training and competition, and this cohort has been characterized in previous work from our group [22,28]. Participants were healthy, free from acute illness and from renal or metabolic disease, and were not taking medication or dietary supplements known to affect the renal handling of water or electrolytes. The study procedures and potential risks were explained to each participant and written informed consent was obtained before enrolment. The study was approved by the Institutional Review Board of King Saud University (Reference KSU-EC: 4/67/352673; approval date 15 May 2015) and was conducted in accordance with the Declaration of Helsinki.

### 2.2. Experimental Design

Players were monitored under their habitual training conditions over six days in September 2015 in Riyadh, Saudi Arabia. The six days were arranged in two blocks of consecutive days (days 1–2 and days 3–6) separated by a four-day interval, reflecting the squad’s scheduled programme. Each session comprised moderate-intensity football training, warm-up, small-sided games, and tactical work, lasting 110 min and conducted from 15:00 to 17:00 [28]. Ambient conditions were characteristic of the Riyadh late summer, with air temperatures routinely exceeding 35 °C. On each day, urine was collected at three standardized time points: the first morning void on waking (MORNING), immediately before the training session (PRE), and immediately after its completion (POST). This yielded up to 18 samples per player and 288 samples in total, of which 287 were available for analysis (one POST sample was not obtained). The morning-to-post progression was designed to span the physiological range of urine concentration, from the rested, typically concentrated first void, through the pre-exercise state, to the post-exercise state, providing the wide within-participant range of creatinine on which the analysis depends.

### 2.3. Urine Sampling and Biochemical Analysis

At each time point, a mid-stream spot-urine sample was collected into a sterile 30 mL container, processed without delay, and stored at −80 °C until batch analysis. Before analysis, samples were thawed, equilibrated to room temperature, and thoroughly mixed. Urinary sodium, potassium, and chloride were measured by integrated multisensor technology (IMT) using ion-selective electrodes; calcium, magnesium, glucose, urea nitrogen, uric acid, total protein, and creatinine were measured by photometric clinical-chemistry assays. All determinations were performed on a Siemens Atellica CH 930 analyzer (Siemens Healthineers, Erlangen, Germany) using the manufacturer’s reagents, calibrators, and quality-control materials, with controls analyzed in each run. Results were reported in the analyzer’s standard units (sodium, potassium, chloride, calcium, magnesium, glucose, and urea nitrogen in mmol·L^−1^; uric acid and creatinine in µmol·L^−1^; total protein in g·L^−1^). Each non-creatinine analyte was additionally expressed as a ratio to urinary creatinine [9,11].

### 2.4. Statistical Analysis

All analyses treated the individual spot-urine sample as the unit of observation, with repeated measures nested within players. Variation in urine concentration was characterized using creatinine and summarized as the median, interquartile range, minimum-to-maximum fold-range, and coefficient of variation, both overall and at each time point. For each non-creatinine analyte, its dependence on urine concentration was quantified in two complementary ways. First, the Spearman rank correlation between the raw analyte concentration and creatinine was computed. Second, the concentration-scaling exponent was estimated as the slope of an ordinary-least-squares regression of the natural logarithm of the analyte concentration on the natural logarithm of creatinine, together with its 95% confidence interval; an exponent of 1 denotes proportional scaling, for which division by creatinine is expected to remove the influence of dilution, whereas exponents below or above 1 indicate that normalization will over- or under-correct, respectively. The adequacy of normalization was then assessed directly by the Spearman correlation between each creatinine-normalized ratio and creatinine: a coefficient near zero indicates that the ratio is independent of urine concentration, whereas a strong negative coefficient indicates that normalization has imposed an artefactual inverse dependence (over-correction). Due to the fact that no universally agreed cutoff for acceptable residual correlation has been established across fields, a ratio–creatinine correlation with an absolute value below 0.3 was adopted as a pragmatic convention for adequate correction, and the influence of this choice was tested by repeating the classification at absolute-r thresholds of 0.2, 0.3, and 0.4. The influence of the normalization choice on inference was evaluated by modelling the within-day (MORNING, PRE, POST) and between-block (days 1–2 versus days 3–6) pattern of each analyte twice, once on raw concentrations and once on creatinine-normalized ratios, using linear mixed-effects models with the time point and block as fixed effects and a random intercept for player. For each analyte, the direction and statistical significance of the pre-to-post change were compared between the raw and normalized models; the analyte was classified as concordant when the two agreed and discordant when they differed in sign or in significance. Skewed variables were natural-log transformed before modelling, and the mixed-effects framework accommodated the single missing observation under a missing-at-random assumption. As a sensitivity analysis, all procedures were repeated after excluding samples with creatinine outside the interpretable range of 0.3–3 g·L^−1^ recommended for biomonitoring [10]. Statistical significance was set at *p* < 0.05, and analyses were performed in IBM SPSS Statistics version 27 (IBM Corp., Armonk, NY, USA).

## 3. Results

### 3.1. Variation in Urine Concentration

Of the 288 scheduled collections, 287 were available for analysis. Urinary creatinine varied 22.7-fold across these samples (median 24,194 µmol·L^−1^; interquartile range 18,252–29,748; range 2520–57,214; coefficient of variation 39%). Concentration increased across the day, the median rising from 22,147 at the morning void to 23,032 pre-training and 26,693 post-training, consistent with progressive fluid loss over the session. Raw analyte concentrations at each time point are summarized in Table 1. Several analytes tracked the concentration change; potassium and chloride rose from morning to pre-training, whereas others did not: urea nitrogen declined across the day, and urinary protein rose only after training, from a median of 0.19 to 0.32 g·L^−1^.

### 3.2. Dependence of Analytes on Creatinine

The analytes differed markedly in how their raw concentrations scaled with creatinine (Table 2). Total protein and glucose scaled almost in proportion to creatinine (scaling exponents 1.03, 95% CI 0.90–1.16, and 0.92, 0.85–0.98) and correlated positively with creatinine in raw form (r = 0.65 and 0.78). The electrolytes and uric acid scaled far less steeply (exponents 0.16–0.49) and their raw concentrations were only weakly, or even negatively, related to creatinine (sodium r = −0.11; chloride −0.04).

### 3.3. Adequacy of Creatinine Normalization

**Figure 1:**
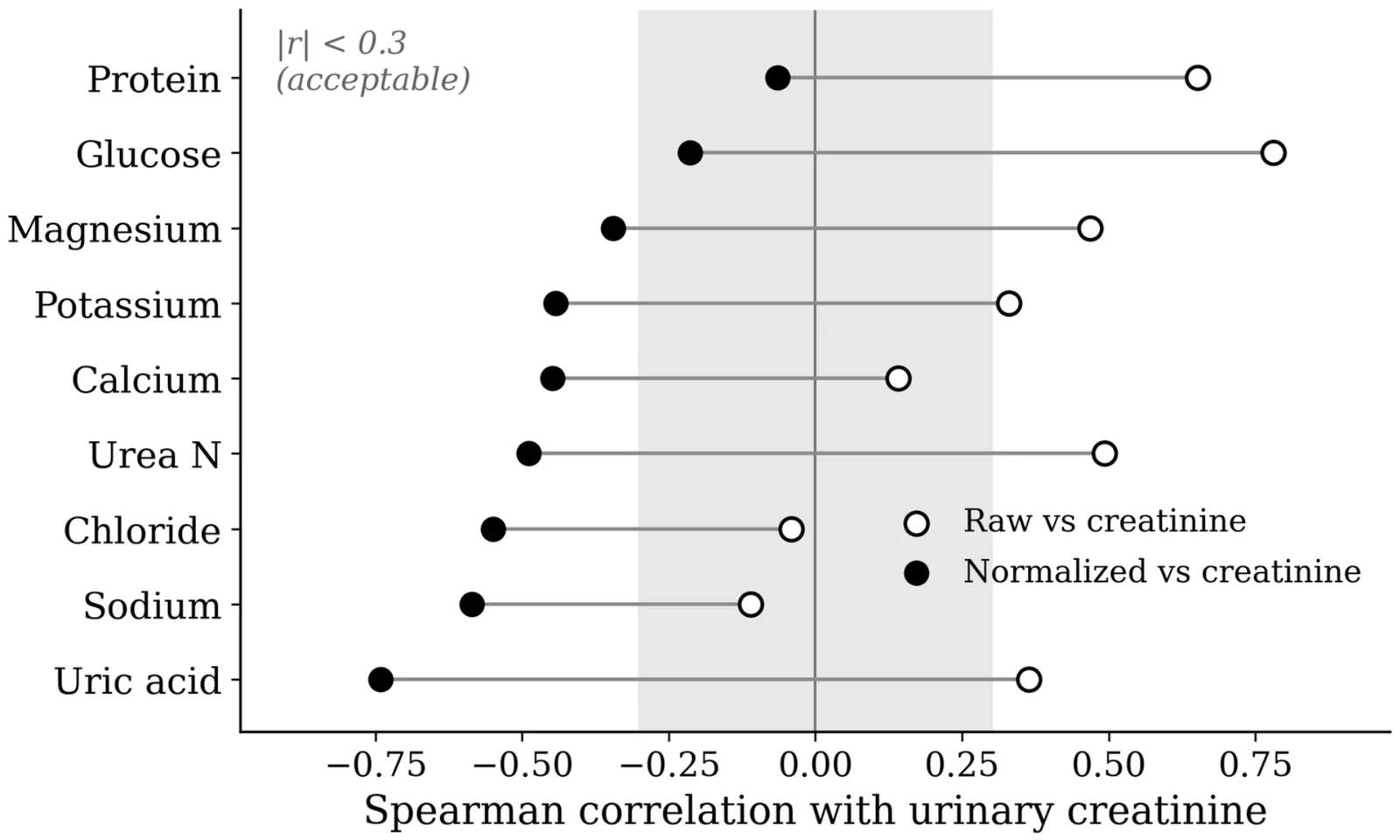
Correlation of each analyte with urinary creatinine before (open circles) and after (filled circles) creatinine normalization. The shaded band marks the region of Spearman r < 0.3, taken here to indicate acceptable, concentration-independent correction; open circles denote the raw analyte versus creatinine and filled circles the creatinine-normalized ratio versus creatinine. For most analytes, normalization shifts the correlation from near zero to strongly negative, indicating an artefactual inverse dependence on urine concentration; only glucose and protein, which scale with creatinine, remain within the acceptable band.

Creatinine normalization removed the dependence on urine concentration for only total protein and glucose, whose normalized ratios were essentially independent of creatinine (r = −0.06 and −0.21; Figure 1). For the remaining seven analytes, normalization over-corrected: it converted a weak raw association into a strong negative correlation between the normalized ratio and creatinine, most markedly for uric acid (r = −0.74), sodium (−0.59), chloride (−0.55), urea nitrogen (−0.49), calcium (−0.45), and potassium (−0.44), with magnesium being intermediate (−0.34).

The classification of adequacy was robust to the threshold used to define it. At r < 0.2 only total protein qualified as adequately normalized; at r < 0.3, total protein and glucose; and at r < 0.4, total protein, glucose, and magnesium. All four electrolytes, together with uric acid and urea nitrogen, were classified as over-corrected at every threshold, so the central finding did not depend on the cut-off chosen.

### 3.4. Effect of Normalization on the Inferred Response

Because normalization altered the concentration dependence of most analytes, it also altered the within-day response that would be inferred (Table 3). For five analytes, potassium, chloride, glucose, total protein, and urea nitrogen, the raw and normalized analyses agreed in the direction and significance of the pre-to-post change. For the remaining four, sodium, magnesium, calcium, and uric acid, they disagreed. Sodium and calcium moved from a non-significant raw change to a significant normalized decline, whereas magnesium and uric acid additionally reversed direction, appearing stable or rising in raw terms but falling significantly once normalized (uric acid raw *p* = 0.47 versus normalized *p* < 0.001).

### 3.5. Sensitivity Analysis

Restricting the analysis to the 182 samples with creatinine within the recommended interpretable range (0.3–3 g·L^−1^) attenuated but did not abolish the over-correction. The residual negative ratio–creatinine correlations were reduced (for example, sodium from −0.59 to −0.29, chloride from −0.55 to −0.28, and urea nitrogen from −0.49 to −0.15) yet remained for the most affected analyte, uric acid (−0.74 to −0.64), while protein and glucose stayed close to zero. Excluding the most extreme urine concentrations therefore lessened, but did not remove, the analyte-specific over-correction.

## 4. Discussion

In these field spot-urine samples, the consequence of creatinine normalization depended entirely on the analyte. Creatinine concentration varied more than twenty-fold across collections, yet dividing by it produced a concentration-independent value for only two of nine analytes, urinary protein and glucose, while leaving the remaining seven, including every electrolyte, carrying a strong artefactual inverse relationship with creatinine. Normalization was therefore not a neutral adjustment that could be applied by default; for most of the panel it replaced one form of concentration dependence with another, and for four analytes it altered the within-day response that would be inferred, in two cases reversing its direction.

This pattern follows directly from how each solute is handled relative to creatinine. The validity of an analyte-to-creatinine ratio rests on the analyte being excreted in proportion to creatinine across the range of urine flow [15]; when it is, as for protein and glucose here (scaling exponents close to one), the ratio cancels the influence of dilution. The electrolytes scaled far less steeply with creatinine because their renal excretion is governed by tubular regulation of sodium, chloride, and the divalent cations rather than tracking water reabsorption. Dividing a weakly concentration-dependent numerator by a strongly concentration-dependent denominator necessarily yields a ratio that falls as the urine concentrates, over-correction, the signature of which, a strong negative correlation between the normalized value and creatinine, was clearly present. That this pattern was attenuated, but not eliminated, when the most extreme urine concentrations were excluded confirms that it is a property of solute handling rather than an artefact of a few very dilute or very concentrated samples.

Several of the analyte-specific patterns were physiologically coherent and illustrate why the normalization choice matters. The pre-training rise and subsequent post-training fall in urinary chloride, sodium, and potassium were consistent with electrolyte intake before training followed by aldosterone-mediated renal conservation as sweating proceeds during exercise in the heat [20]; the parallel decline in urea nitrogen reflects similarly regulated, concentration-independent handling; and the rise in total protein only after training was the expected signature of transient, exercise-induced proteinuria [29]. Due to the fact that these solutes were regulated largely independently of urine flow and creatinine, expressing them as creatinine ratios distorts rather than clarifies these responses most strikingly for uric acid and calcium, whose apparent within-day direction reversed after normalization. Interpreting such reversals as physiology rather than artefact would lead to materially wrong conclusions about how an athlete was handling water and electrolytes.

These observations were consistent with long-standing cautions from clinical chemistry, biomonitoring, and urinary metabolomics, where creatinine excretion was known to vary with muscle mass, diet, and glomerular filtration and where simple creatinine ratios can misrepresent excretion when those conditions are not met [10,15,16]; in athletes specifically, lean mass alone biases creatinine-referenced hydration indices [17]. The present data add an internal check that strengthens this interpretation. The one analyte in the panel whose creatinine ratio is an established, clinically validated measure, urinary protein, reported as the protein-to-creatinine ratio as a surrogate for quantified proteinuria [30], was also the one that normalized most cleanly. That the analysis endorsed the analyte known to belong in a creatinine ratio while flagging the electrolytes that have no comparable validation indicates that the over-correction detected here is a property of the solutes themselves, not an artefact of the method.

The practical implication is pointed. In the monitoring of athletes exercising in the heat, the analytes of primary interest are precisely the electrolytes, sodium and chloride above all, and these were exactly the analytes for which creatinine normalization performed worst [7]. A creatinine-referenced electrolyte value obtained in this setting will tend to read low when the urine is concentrated, which is to say during the very dehydration it is intended to detect, so the correction can move the measurement in the wrong direction at the moment it matters most.

For sports-science practice, three concrete recommendations follow. First, raw spot-urine electrolyte concentrations should not be interpreted without reference to urine concentration, and creatinine ratios should not be assumed to supply that reference for electrolytes. Second, where feasible, an independent concentration marker, such as urine specific gravity measured by handheld refractometry, which is inexpensive and field-portable, should also be measured, and electrolytes should be interpreted relative to it rather than to creatinine. Third, whenever creatinine normalization is used, its adequacy for the analyte in question should be verified, for example, by the simple residual-correlation check applied here, and the normalization method should always be reported. Applied uncritically, creatinine-normalized electrolyte ratios could cause a dehydrating athlete to be judged adequately replete, delaying rehydration and, in extreme heat, increasing the risk of heat-related illness; these are the misconceptions the present findings caution against.

These implications extend beyond football. The same creatinine-referenced ratios are used to estimate population sodium intake from spot urine [21,22], to interpret occupational and environmental biomonitoring [10,16], and to normalize urinary metabolomic profiles for biomarker discovery [11,12,13,14]. In each application, an analyte-specific over-correction of the kind shown here could bias comparisons between more- and less-concentrated samples, for example, between dehydrated and euhydrated athletes, or between clinical groups differing in muscle mass or renal function.

The findings also bear on the wider question of how urinary data should be adjusted for dilution, for which three approaches are in common use. Creatinine normalization was simple and near-universal but, as shown here, assumes a proportional scaling that holds only for some analytes. Osmolality- or specific-gravity-based adjustment corrects for total solute concentration or density directly, does not depend on the renal handling of any single reference solute, and has been reported to improve statistical discrimination in urinary metabolomics [12,13]. Probabilistic quotient normalization, which scales each sample to a reference profile, suits broad untargeted metabolomic data but is less interpretable for a small targeted panel and can itself be distorted by a few large, concentration-dependent signals [11,14]. For heat-stressed athlete urine, in which electrolytes are the primary interest and concentration varies widely, an osmolality- or specific-gravity-based adjustment is therefore likely to be the most appropriate, with creatinine reserved for analytes, such as total protein, demonstrably shown to scale proportionally with it.

Several limitations qualify these findings and define the next steps. First, and most important, no independent measure of urine concentration, osmolality or specific gravity, was collected, so although we can demonstrate that creatinine normalization introduces a concentration-dependent bias and can change inferred responses, we cannot quantitatively compare its performance against an alternative adjustment. The direct test was straightforward and should be the immediate follow-up: measuring urine osmolality and specific gravity on the same spot samples alongside creatinine and, for each analyte, comparing the residual correlation of each adjusted value with the reference of concentration; the adjustment yielding values most independent of concentration and most consistent with a criterion of hydration such as pre-to-post body-mass change would be preferred. Second, the cohort comprised sixteen young male professional soccer players from a single squad in one hot climate, so the results may not extend to women, to other age groups, to other sports, or to temperate conditions, and confirmation across these groups is required. Third, fluid intake, dietary electrolyte intake, body-mass change, and heat-acclimatization status were not recorded; these were determinants of urinary electrolyte excretion and could have contributed both to the between-block differences and to the scaling relationships observed, and future designs should record them as covariates. Finally, the observational field design precludes separating dilution from genuine changes in excretion at the individual level. These constraints bound the interpretation but did not affect the central, internally validated observation that creatinine normalization behaves differently across analytes.

Based on these limitations, future work should collect osmolality or specific gravity alongside creatinine so that competing dilution adjustments can be compared directly against a reference [12,13,14] and should evaluate analyte-specific corrections, such as a power-function adjustment that accommodates each solute’s own scaling with concentration, against the conventional ratio. Pairing urinary markers with body-mass change and an acclimatization marker such as aldosterone would allow the physiological interpretation of electrolyte conservation to be tested directly. Finally, replication in larger, multi-site cohorts, including female athletes and a range of sports and environmental conditions, would establish the generality of the analyte-specific pattern reported here.

## 5. Conclusions

In field spot-urine samples from soccer players training in extreme heat, creatinine normalization behaved very differently across analytes: it was adequate for total protein and glucose, which scale proportionally with creatinine, but over-corrected all four electrolytes as well as uric acid and urea nitrogen, and for four of nine analytes it changed or reversed the within-day response that would be inferred. This classification was robust to the threshold used to define adequacy. For hydration monitoring in hot-environment team sports, where electrolytes are the analytes of primary interest, creatinine normalization should therefore not be assumed adequate, and spot-urine electrolytes should be interpreted against an independent measure of urine concentration such as osmolality or specific gravity. The simple, internally validated approach used here, anchored by a clinically established protein-to-creatinine ratio that behaved as expected, offers a practical way to test the adequacy of normalization in other datasets. The principal priorities for future research are the direct comparison of creatinine, osmolality, and specific-gravity adjustment on identical samples against a hydration criterion and replication in women, in other sports, and across a range of climates.

## Figures and Tables

**Table 1:** Raw urinary analyte concentrations at each collection time point (median with interquartile range; n = 16 players).

Analyte	Unit	Morning	Pre-Training	Post-Training
Sodium	mmol/L	105.5 (60.0–146.8)	129.0 (75.3–188.3)	115.0 (66.5–160.0)
Potassium	mmol/L	47.1 (32.0–59.4)	73.6 (53.3–107.6)	73.8 (60.2–94.4)
Chloride	mmol/L	86.5 (59.5–140.0)	195.0 (124.5–259.0)	174.0 (116.5–233.5)
Calcium	mmol/L	2.23 (1.61–3.50)	2.69 (1.77–4.64)	2.60 (1.37–3.67)
Magnesium	mmol/L	6.12 (4.24–7.86)	5.10 (3.22–6.85)	5.18 (3.65–6.74)
Glucose	mmol/L	1.23 (0.83–1.47)	1.06 (0.84–1.36)	1.46 (1.07–1.82)
Urea N	mmol/L	486.9 (347.9–609.2)	426.7 (297.0–544.9)	381.5 (283.2–481.4)
Uric acid	µmol/L	2158 (1902–2679)	2138 (1827–2643)	2256 (1863–2695)
Total protein	g/L	0.19 (0.15–0.23)	0.19 (0.14–0.24)	0.32 (0.23–0.46)
Creatinine	µmol/L	22,147 (16,884–28,596)	23,032 (17,668–28,617)	26,693 (20,300–33,197)

Values are median (interquartile range). Units are those reported by the analyzer (see Section 2.3): sodium, potassium, chloride, calcium, magnesium, glucose, and urea nitrogen in mmol·L^−1^; uric acid and creatinine in µmol·L^−1^; total protein in g·L^−1^.

**Table 2:** Dependence of each urinary analyte on urine concentration and the effect of creatinine normalization.

Analyte	Scaling Exponent (95% CI)	Raw vs. Creatinine, r	Ratio vs. Creatinine, r	Normalization
Total protein	1.03 (0.90 to 1.16)	0.65	−0.06	Adequate
Glucose	0.92 (0.85 to 0.98)	0.78	−0.21	Adequate
Magnesium	0.67 (0.56 to 0.78)	0.47	−0.34	Over-corrected
Potassium	0.49 (0.36 to 0.61)	0.33	−0.44	Over-corrected
Calcium	0.36 (0.20 to 0.52)	0.14	−0.45	Over-corrected
Urea N	0.67 (0.59 to 0.75)	0.49	−0.49	Over-corrected
Chloride	0.25 (0.09 to 0.41)	−0.04	−0.55	Over-corrected
Sodium	0.16 (−0.02 to 0.34)	−0.11	−0.59	Over-corrected
Uric acid	0.35 (0.29 to 0.42)	0.36	−0.74	Over-corrected

Scaling exponent = Slope of log(analyte) on log(creatinine); a value near 1 indicates proportional scaling. r = Spearman correlation. A ratio-versus-creatinine r near zero indicates adequate correction; a strong negative value indicates over-correction. Analytes are ordered by ratio-versus-creatinine r. All quantities in this table are dimensionless (correlation coefficients and log–log slopes).

**Table 3:** Within-day (pre-to-post-training) response of each analyte inferred from raw versus creatinine-normalized values.

Analyte	Raw: Direction (*p*)	Normalized: Direction (*p*)	Agreement
Potassium	Post < Pre (<0.001)	Post < Pre (<0.001)	Concordant
Chloride	Post < Pre (<0.001)	Post < Pre (<0.001)	Concordant
Glucose	Post > Pre (<0.001)	Post > Pre (0.003)	Concordant
Total protein	Post > Pre (<0.001)	Post > Pre (0.002)	Concordant
Urea N	Post < Pre (<0.001)	Post < Pre (<0.001)	Concordant
Sodium	Post < Pre (0.062)	Post < Pre (0.028)	Discordant
Calcium	Post < Pre (0.099)	Post < Pre (0.047)	Discordant
Magnesium	Post > Pre (0.269)	Post < Pre (0.003)	Discordant
Uric acid	Post > Pre (0.472)	Post < Pre (<0.001)	Discordant

Direction and *p*-value of the pre-to-post change from linear mixed-effects models. “Discordant” denotes a difference between raw and normalized analyses in the sign or the statistical significance of the change.

## Data Availability

The data are available from the corresponding author upon reasonable request.
